# Oxidative Stress and Its Significant Roles in Neurodegenerative Diseases and Cancer

**DOI:** 10.3390/ijms16010193

**Published:** 2014-12-24

**Authors:** Raynoo Thanan, Shinji Oikawa, Yusuke Hiraku, Shiho Ohnishi, Ning Ma, Somchai Pinlaor, Puangrat Yongvanit, Shosuke Kawanishi, Mariko Murata

**Affiliations:** 1 Department of Biochemistry, Faculty of Medicine, Khon Kaen University, Khon Kaen 40002, Thailand; E-Mails: rothanan@yahoo.com (R.T.); puangrat@kku.ac.th (P.Y.); 2 Liver Fluke and Cholangiocarcinoma Research Center, Faculty of Medicine, Khon Kaen University, Khon Kaen 40002, Thailand; E-Mail: mrsomchaip@yahoo.com; 3 Department of Environmental and Molecular Medicine, Mie University Graduate School of Medicine, 2-174 Edobashi, Tsu, Mie 514-8507, Japan; E-Mails: s-oikawa@doc.medic.mie-u.ac.jp (S.O.); y-hiraku@doc.medic.mie-u.ac.jp (Y.H.); 4 Faculty of Pharmaceutical Sciences, Suzuka University of Medical Science, Suzuka, Mie 513-8670, Japan; E-Mails: shiho-o@suzuka-u.ac.jp (S.O.); kawanisi@suzuka-u.ac.jp (S.K.); 5 Faculty of Nursing Science, Suzuka University of Medical Science, Suzuka, Mie 513-8670, Japan; E-Mail: maning@suzuka-u.ac.jp; 6 Department of Parasitology, Faculty of Medicine, Khon Kaen University, Khon Kaen 40002, Thailand

**Keywords:** oxidative stress, neurodegenerative diseases, cancer, lipid peroxidation, oxysterol, carbonyl proteins, protein damage, DNA damage, stem cells

## Abstract

Reactive oxygen and nitrogen species have been implicated in diverse pathophysiological conditions, including inflammation, neurodegenerative diseases and cancer. Accumulating evidence indicates that oxidative damage to biomolecules including lipids, proteins and DNA, contributes to these diseases. Previous studies suggest roles of lipid peroxidation and oxysterols in the development of neurodegenerative diseases and inflammation-related cancer. Our recent studies identifying and characterizing carbonylated proteins reveal oxidative damage to heat shock proteins in neurodegenerative disease models and inflammation-related cancer, suggesting dysfunction in their antioxidative properties. In neurodegenerative diseases, DNA damage may not only play a role in the induction of apoptosis, but also may inhibit cellular division via telomere shortening. Immunohistochemical analyses showed co-localization of oxidative/nitrative DNA lesions and stemness markers in the cells of inflammation-related cancers. Here, we review oxidative stress and its significant roles in neurodegenerative diseases and cancer.

## 1. Introduction

**Figure 1:**
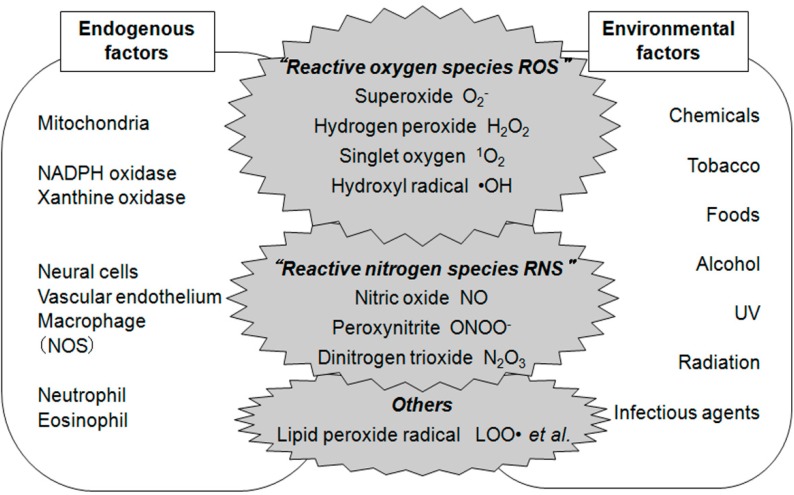
Reactive oxygen species and reactive nitrogen species (ROS and RNS) and their sources from endogenous and environmental factors.

Oxygen is one of the most prevalent elements on Earth and is essential for energy production by the living organisms known as “aerobes”. However, oxygen is also an initiator of free radical generation, which can injure the living organism. Antioxidant systems are adapted in aerobic organisms for protection against free radical toxicity. Oxidative stress is a situation that introduces a high production of oxidants or a low level of antioxidants, which results in an imbalance between oxidant and antioxidant systems causing free radical damage. Figure 1 shows the source of reactive oxygen species and reactive nitrogen species (ROS and RNS). Oxidative stress can be extrinsically induced by environmental factors such as chemicals, UV light, infectious organisms and intrinsically by endogenous factors such as the electron transport chain in mitochondria, some enzyme activities (for example: NADH oxidase and nitric oxide synthase (NOS)) and respiratory bursts from inflammatory cells.

**Figure 2:**
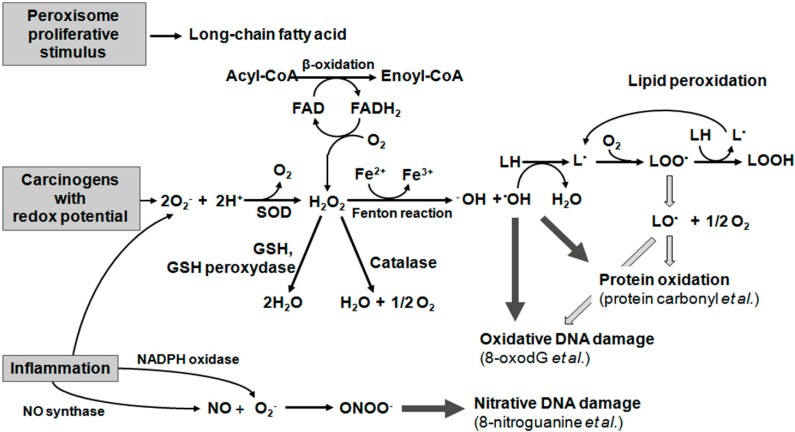
Oxidative damage to biomolecules.

Oxidative stress is critical to the etiology of many “oxidative stress related diseases”, especially neurodegenerative diseases and cancers. Inflammation induces ROS and RNS production via respiratory bursts and inflammatory cytokines, which can activate many oxidant generating enzymes such as inducible nitric oxide synthase (iNOS), cyclooxygenase 2 (COX2), myeloperoxidase (MPO) and eosinophil peroxidase (EPO). Respiratory burst oxidase generates superoxide (O_2_^•−^) via the one electron-reduction of oxygen by NADPH, with a secondary production of hydrogen peroxide (H_2_O_2_), hydroxyl radical (•OH), hypochlorous acid (HOCl), and other activated forms of oxygen [1]. In contrast, RNS including nitric oxide (NO) are generated mainly under inflammatory conditions via the expression of iNOS. NO reacts with O_2_^•−^ to form highly reactive peroxynitrite (ONOO^−^) [2]. Figure 2 shows the general mechanisms of oxidative damage to biomolecules, which results in the dysfunction of the biomolecules and interference with the signaling pathways, leading to oxidative stress-induced diseases.

Oxidative damage to lipids causes lipid peroxidation and oxysterol formation, which mainly localize in the cellular membrane resulting in a loss of membrane property. Their reactive end products can consequently damage other molecules. Proteins are mainly functional biomolecules that drive cellular activity. Oxidative damage to proteins may result in protein dysfunction. DNA damage also plays significant roles in not only mutations, but also genetic instability and epigenetic changes. Moreover, many kinds of oncogenes and tumor suppressor genes can be altered by oxidative stress. Therefore, oxidative stress induces disease through the oxidative modification of biomolecules and the alteration of signaling pathways leading to dysregulation of cell cycles, interference with cellular metabolism, genetic instability, epigenetic change and mutation.

## 2. Oxidative Damage to Lipids

### 2.1. Lipid Peroxidation

Lipid peroxidation is a complex reaction of free radical attacks on polyunsaturated fatty acids (PUFA) which are conjugated with the glycerol or sphingosine of phospholipids in the lipid bilayer of biological membranes resulting in a chain reaction due to unstable intermediates (LOO• and LOOH) that can lead to a loss of membrane properties and reactive end products, which can further interact with other molecules causing protein and DNA damage. Many types of reactive end products from lipid peroxidation have been investigated including malondialdehyde, 4-hydroxy-2-nonenal (HNE), acrolein and isoprostanes. Among these, HNE and malondialdehyde have been the most intensively studied as they are highly toxic molecules due to their high electrophilic properties and easily react with proteins and DNA [3,4] resulting in the formation of protein carbonyls and etheno DNA adducts, respectively. Moreover, malondialdehyde is the most common biomarker for oxidative stress due to its stability, and useful detection methods have already been developed [5]. The termination of lipid peroxidation can occur by two radicals reacting to form a nonradical and oxygen, and by reaction with a chain-breaking antioxidant (Vitamin E) inserted in the lipid bilayer structure with the collaboration of Vitamin C and other antioxidant systems in cytoplasm [4].

### 2.2. Oxysterols

Oxysterols are oxygenated cholesterol derivatives that are generated by enzyme-specific reactions mediated mainly by cytochrome P450 or by non-specific reactions involving ROS and RNS. The chemical structures of oxysterols vary depending on the number and position of oxygenated functional groups including keto-, hydroxyperoxy- and epoxy forms [6]. Oxysterols act as intermediates in the cholesterol catabolism, especially in bile acid synthesis, which is subsequently eliminated from the body. However, the excessive generation of oxysterols was found to be involved in the pathologic effects of many diseases including neurodegenerative diseases and cancers [6,7]. Oxidative stress induces the formation of oxysterols which are oxygenated mainly in the C-6 and C-7 positions such as 7α-hydroperoxycholesterol, 7-ketocholesterol and 7α/β-hydroxycholesterol. Among them, 7α-hydroperoxycholesterol and 7β-hydroxycholesterol can be used as oxidative stress biomarkers [8]. Previous studies have suggested that not only oxidative stress can induce the formation of oxysterols, but that both enzymatic and non-enzymatic oxysterols can also induce oxidative stress via the induction of free radical generation, pro-inflammatory cytokine production, glutathione suppression and COX2 expression [9,10,11]. Oxysterols have been ascribed a number of important roles in connection with cholesterol turnover, atherosclerosis, apoptosis, necrosis, inflammation, and immunosuppression [12].

## 3. Oxidative Damage to Protein

### Carbonyl Protein as a Potential Oxidative Damage Biomarker

Free radicals that are overproduced in both oxidative and nitrative stresses can also damage proteins. The oxidative and nitrative modifications of proteins by ROS and RNS, respectively, are implicated in the etiology or progression of disorders and diseases, including chronic inflammatory diseases, such as cancer, atherosclerosis, rheumatoid arthritis, ischemia reperfusion injury, and acute pancreatitis, aging and age-related neurodegenerative disorders, among which the most prevalent is Alzheimer’s disease [13,14,15,16,17]. These are all chronic inflammatory diseases. ROS damage proteins with oxidative modifications such as sulphur oxidation of methionine and cysteine, protein hydroxides and carbonyl derivatives from the oxidation of amino-acid side chains (lysine, arginine, proline, threonine, cysteine and histidine) [18]. Cysteine and methionine are particularly prone to oxidative attack by almost all ROS, leading to the formation of Cys-SO_x_H (x = 1, 2 or 3) and methionine sulfoxide. RNS induce protein nitration such as 3-nitrotyrosine, an inflammation-associated marker. Carbonylated proteins are defined as oxidation-damaged proteins that induce carbonyl (ketone (–CO–) or aldehyde (–COH) groups) formation in amino acid side chains. They are generated from direct oxidative damage to proline, arginine, lysine and threonine residues resulting in glutamic semialdehyde (from proline and arginine), aminoadipic semialdehyde (from lysine) and 2-amino-3-ketobutyric acid (from threonine) [19]. Moreover, protein carbonyls are also generated from the secondary reactions of reactive carbonyl derivatives end products from lipid peroxidation (HNE, malondialdehyde, 2-propanyl and 4-oxo-nonenal) and glycation/glycosidation (ketoamines, ketoaldehydes and deoxyosones) reactions into cysteine, histidine and lysine residues [18,20]. Oxidative damage to proteins is irreversible and unrepairable, whereas damaged proteins can activate proteosomes for degradation of the oxidized proteins [21]. However, several oxidized proteins were found to accumulate in cells via aggregations of those proteins resulting in interference with proteosome functions [21]. Carbonylated proteins are relatively difficult to induce compared with the SH oxidation of cysteine and methionine, and elevated levels of carbonylation are thought to be a sign not only of oxidative stress, but also of disease-derived protein dysfunction [22]. Therefore, carbonylated proteins can be used as oxidative stress biomarkers according to their special properties including irreversibility, unrepairability, stability in physical condition and induction of protein aggregation. Levine and coworkers developed a highly sensitive method for the detection of carbonylated proteins involving derivatization of the carbonyl group with 2,4-dinitrophenylhydrazine (DNPH), which leads to a 2,4-dinitrophenyl (DNP) hydrazone adduct [23]. The analysis of carbonylated protein with DNPH reaction and specific antibody for DNP hydrazone by immunoblot in 2D gel electrophoresis (2D-oxyblot) was pioneered by Nakamura and Goto [24]. The DNP hydrazone adduct was also measured by spectrophotometry, HPLC, ELISA, slot blotting and Western blot analysis as already summarized by Dalle-Donne *et al.* [19]. In addition, 2D-oxyblot and tandem mass spectrometry (MS/MS) can be used for the identification and detection of carbonylated regions of carbonylated proteins in various types of sample [25,26,27]. Meanwhile, enrichment of DNP derivatized proteins by affinity chromatography followed by identification of oxidized sites using MS/MS mass spectrometry methods have also been applied [28,29,30,31,32]. Thus, the identification of carbonylated proteins and oxidized sites can be used for investigation of the significant roles of carbonylated proteins in oxidative stress-related diseases.

## 4. Oxidative Damage to DNA

### 4.1. DNA Damage Formation from Oxidative Modification

The oxidatively induced DNA damage typically associated with ROS are apurinic/apyrimidinic (AP) DNA sites, oxidized purines and pyrimidines, single strand and double strand DNA breaks. Kryston *et al.* reviewed the role of oxidative stress and DNA damage in human malignancies, and suggested the idea of using several oxidatively generated DNA lesions like 8-oxo-7,8-dihydro-2'-deoxyguanine (8-oxodG), also known as 8-hydroxydG (8-OHdG) , thymine glycol, AP sites and oxidatively generated clustered DNA lesions (OCDLs) as novel biomarkers of oxidative stress [33]. OCDLs are complex DNA damage (tandem lesions, intra- and interstrand cross-links, DNA–protein cross-links) induced by •OH and one-electron oxidants [34]. The clustered DNA lesions are refractory to repair, thus promoting mutation and genome instability through chromosome breakage [35]. On the other hand, the DNA lesion with physiological relevance to neural cells are single strand DNA breaks, which arise from the disintegration of the sugar phosphate backbone of DNA following oxidative attack by ROS [36]. Double strand DNA breaks are the most genotoxic DNA lesions and the unrepaired lesions can lead to neural cell death, leading to neurodegeneration. Single strand and double strand DNA breaks are typically detected in the brain in Alzheimer’s disease and other neurodegenerative diseases. Also, the accumulation of oxidative DNA damage may be linked with age-associated neurodegenerative disorders Alzheimer’s disease, Parkinson’s disease and amyotrophic lateral sclerosis. ROS generate 8-oxodG, which can be used as an indicator of oxidative DNA damage in relation to oxidative stress-driven diseases [37,38]. 8-OxodG formation can be detected in cellular DNA and body fluids through two main approaches; specific antibody- and chromatography-based methods [39]. There is growing clinical interest in the measurement of urinary 8-oxodG as a mean to determine the role of oxidative stress in disease and to evaluate intervention strategies [40]. In both human malignancies and neurodegenerative diseases, 8-oxodG is detected in tissues and body fluids such as serum/plasma, cerebral spine fluid and urine because this lesion exists at steady high levels in genomic and mitochondrial DNA. Therefore, 8-oxodG is a suitable biomarker for oxidative stress.

### 4.2. DNA Damage Formation from Nitrative Modification

Nitric oxide (NO), a primary initiator of RNS, is generated specifically during inflammation via iNOS in inflammatory and epithelial cells [38,41]. Overproduction of NO participates with superoxide anion in the generation of peroxynitrite (ONOO^−^), which is a more reactive molecule and can form nitrative DNA lesion, 8-nitroguanine [42,43]. Therefore, 8-nitroguanine is a specific biomarker for inflammation-related DNA damage. 8-Nitroguanine in RNA was found to be much more stable than in DNA, which rapidly depurinates to release the modified base [44]. High accumulation of 8-nitroguanine in DNA and RNA can be detected by specific antibodies [45] and chromatography-based methods [46]. Therefore, 8-nitroguanine is a suitable biomarker for inflammation-related oxidative stress.

### 4.3. 8-OxodG and 8-Nitroguanine Are Potential Mutagenic DNA Lesions

Human oxyguanine glycosylase 1 (hOGG1), a DNA glycosylase and base excision repair (BER) enzyme, removes 8-oxoG from genomic/mitochondrial DNA [47]. Moreover, several molecules that are involved in mismatch repair (MMR) and NER also play important roles in preventing mutations due to 8-oxodG formation [48,49,50]. Recently, Rodriguez *et al.* demonstrated, in work with oligonucleotides containing an 8-oxodG, that this lesion can induce template switching, thereby bypassing the damaged base [51]. However, deficiency in the repair of nuclear and mitochondrial DNA damage is linked to several neurodegenerative disorders and cancers [52,53]. Therefore, high formation of 8-oxodG is a potentially mutagenic DNA lesion that leads to the transversion of G:C to T:A (G→T transversion) via mis-complementation with deoxyadenosine-5'-triphosphate (dATP) during DNA replication [54].

Apurinic sites are formed in DNA via the spontaneous depurination of 8-nitroguanine, leading to G→T transversion through the incorporation of dATP during DNA replication [55,56]. DNA polymerase η and κ were involved in the incorporation of dATP opposite 8-nitroguanine during DNA synthesis in a cell-free system associated with trans-lesion DNA synthesis leading to the G→T transversion [57]. Moreover, DNA polymerase ζ can efficiently bypass apurinic/apyrimidinic sites by extending from nucleotides inserted opposite the lesion by other DNA polymerases [58]. DNA polymerase was hypersensitive to nitrative stress, and trans-lesion DNA synthesis past apurinic sites mediated by this polymerase contributes to extensive point mutations [59]. The specific repair system of 8-nitroguanine in DNA has not yet been discovered, whereas the apurinic site repair system could be corroborated. Therefore, 8-nitroguanine is a potential mutagenic DNA lesion involved in inflammation-mediated carcinogenesis.

Interestingly, exome sequencing identified G→T transversion mutations in several genes including KRAS and TP53 in liver fluke-related bile duct cancers [60]. This point mutation was also frequently found in KRAS mutation in colorectal cancers [61]. p53 mutation in lung cancers implies an excess of G→T transversion, which is a molecular signature of tobacco smoke mutagens in smoking-associated lung cancers [62]. It is potentially a potent weapon for predicting cancer, if mutation-prone DNA lesions such as 8-oxodG and 8-nitroguanine could be identified in genome scales.

## 5. Oxidative Stress and Neurodegenerative Diseases

### 5.1. Lipid Oxidation in Neurodegenerative Diseases

The brain consumes a lot of oxygen and antioxidants, and therefore oxidative stress can easily occur in the brain. Oxidative damage to lipids is important in neurodegenerative disease development because polyunsaturated fatty acids are abundant in the lipid bilayer of the brain [63]. Increases in lipid peroxidation end products were found in Alzheimer’s, Parkinson’s, and Huntington’s disease [4]. About 25% of brain lipids are cholesterol, which acts as an electrical insulator along the length of large axons [64]. Oxysterol formations are the maintenance mechanism for the balance between cholesterol synthesis and efflux. Circulating 24-hydroxycholesterol (24-HC) is a major enzymatic oxysterol (from CYP46A1 activity) produced by the brain [65]. The cholesterol levels in serum or plasma do not correlate with Alzheimer’s disease, whereas the 24-HC level is significantly elevated and correlated with multiple proteins such as amyloid precursor proteins and amyloid β (Aβ) in patients, suggesting that Alzheimer’s disease may impact cholesterol homeostasis [66]. Additionally, circulating 27-hydroxycholesterol, an oxysterol secreted from macrophages, has the capacity to pass through the blood brain barrier into the brain and is able to suppress expression of a memory protein (Arc), suggesting that it may also be important for the generation of β-amyloid peptides [64]. Therefore, these lipid oxidation end products can be used as diagnostic markers in neurodegenerative diseases.

### 5.2. Carbonyl Protein in Neurodegenerative Diseases

Proteins vulnerable to ROS could be categorized into diverse functional classes such as nucleocytoplasmic transport, immunity and defense, energy metabolism, ubiquitination-proteasome pathway, neurotransmitter and purine metabolism [67]. The accumulation of highly crosslinked protein aggregates leads to further oxidant formation, damage to macromolecules, and, finally, to apoptotic cell death. The age-dependent accumulation of carbonyl proteins in the brain may be related with age-dependent susceptibility to neurodegenerative diseases [68]. The highly oxidized lipofuscin accumulates during aging, and lipofuscin formation may cause impaired lysosomal and proteasomal degradation, metal ion accumulation, increased reactive oxygen species formation, and apoptosis [69]. α-Synucleinopathies include Parkinson’s disease, dementia with Lewy bodies, and multiple system atrophy. Surgucheva *et al.* demonstrated the colocalization of oxidized-γ-synuclein with phospho-α-synuclein in the brain and the neuronal accumulation of aberrant γ-synuclein in neurodegenerative diseases, suggesting that γ-synuclein plays an important role in α-synuclein aggregation [70]. Carbonylated proteins are highly accumulated in the Alzheimer’s disease brain and are localized to paired helical filaments (PHF) and amyloid plaques, hallmarks of Alzheimer’s disease, suggesting that these protein modifications may play a causal role in the progression of the neurodegenerative disease [71]. Since oxidative damage may result in impaired function, protein oxidative damage may have important consequences for the nervous system, resulting in abnormal glycolysis and energy metabolism, abnormal responses to protein folding and oxidative stress responses, cytoskeletal abnormalities, and impaired protein degradation [72]. Such carbonylated proteins were detected in an animal model of the neurotoxic effects of 1-bromopropane (1-BP), suggesting that 1-BP-induced hippocampal damage involves oxidative stress, loss of ATP production, neurotransmitter dysfunction and inhibition of the ubiquitination-proteasome system [67]. Our series of reports found that carbonylated heat shock protein 70 (HSP70) was detected by 2D-oxyblot and MALDI-TOF/TOF analysis in animal models of kainic acid-induced status epilepticus [73] and ischemia-reperfusion [25,26]. HSP70 is known to protect cells from oxidative stress and stabilize the lysosomal membrane, and therefore our results and previous studies suggest that carbonylated HSP70 may induce autophagy of neurons via the lysosomal degradation pathway [74]. We then identified several carbonylated proteins that correlated with neurodegenerative disease via their protein dysfunction contributions to decreased neuron activity, energy supply and induction of neuron cell death via autophagy and prolongation of oxidative stress.

### 5.3. DNA Damage in Neurodegenerative Diseases

The accumulation of DNA damage and decline in DNA repair is a general phenomenon in the aging brain. The age-related accumulation of DNA damage in specific populations of neurons may be among the most important molecular mechanisms triggering the onset of neurodegenerative diseases [75]. Elevated levels of oxidative lesions were reported in the neurons of amyotrophic lateral sclerosis patients, and damage to mitochondrial DNA has been documented in Parkinson’s disease [76].

The major manifestation of metal toxicity is aging and age-related neurological diseases. Compelling evidence has etiologically linked abnormal brain metal accumulations with aging and various neurological disorders including Alzheimer’s disease, Parkinson’s disease, amyotrophic lateral sclerosis, stroke, and dietary or occupational exposure [77]. Halliwell and his colleagues proposed that metal ion release, in the presence of DOPA and dopamine, may be an important mechanism of neurotoxicity, e.g., in Parkinson’s disease [78]. Interestingly, we demonstrated that Mn(II) enhanced the cell death of dopaminergic neuron model PC-12 cells induced by dopamine via oxidative DNA damage [79]. Also, we showed that 6-hydroxydopamine-induced apoptosis and the formation of 8-oxodG in neuroblastoma SH-SY5Y cells were inhibited by iron or copper chelators, suggesting the involvement of iron and copper in intracellular oxidatively generated damage to DNA, a stimulus for initiating apoptosis [80]. Our *in vivo* studies showed oxidative/nitrative DNA damage in relation to neurotoxicity in the brain of mice exposed to arsenic [81]. Additionally, pro-oxidant metals inhibit DNA repair pathways [77], and therefore, abnormal brain metal accumulations create an imbalance between genome damage and repair, and the resulting persistent accumulation of damage contributes to neuronal dysfunction and cell death.

As another source of ROS, abnormal protein aggregations may be an important factor. The aggregation of abnormal protein components leads to their selective loss in disorders such as Alzheimer’s disease (aggregations of Aβ and hyperphosphorylated tau proteins leading to neurofibrillary tangles), Parkinson’s disease (aggregation of α-synuclein leading to intracellular Lewy bodies formation) and Huntington’s disease (aggregation of Huntington protein) [82]. Aggregation of these abnormal proteins further induces oxidative stress via induction of mitochondria dysfunction and ROS production resulting in mitochondria DNA damage that triggers apoptosis [83].

## 6. Oxidative Stress and Cancers

### 6.1. Lipid Oxidation in Cancers

Our previous studies demonstrated significantly higher plasma levels of isoprostanes and malonaldehyde in cholangiocarcinoma patients and subjects with liver fluke infection in comparison with healthy subjects [84]. Interestingly, etheno DNA adducts, which are potential mutagenic lesions, were increased in the leukocyte DNA and excretory urine of liver fluke (*Opisthorchis viverrini*)-infected subjects and cholangiocarcinoma patients, and etheno DNA adduct levels were also significantly positively correlated with isoprostanes, malondialdehyde and nitrate/nitrile levels in plasma [84,85]. Moreover, large numbers of these lesions were seen in the cholangiocyte cells of parasite-infected hamsters and the cancer cells of cholangiocarcinoma in the livers of an *O. viverrini*-induced cholangiocarcinoma in animal model [86]. The series of studies suggested that oxidative stress induced by liver fluke infection contributes to lipid peroxidation and DNA damage, which may synergistically result in mutation and cholangiocarcinoma development. Oxysterol binding proteins (OSBPs) were significantly over expressed in liver fluke-associated cholangiocarcinoma in an animal model and in clinical samples with different isoforms (OSBPL8 and OSBPL7 in a hamster model and OSBP2 and OSPBL8 in blood samples from cancer patients) [87]. Cholestan-3β,5α,6β-triol (Triol) and 3-keto-cholest-4-ene (3K4) levels were significantly higher in the livers of hamsters with liver fluke-induced cholangiocarcinoma and functional analysis of these oxysterols suggested their roles in the induction of oxidative stress leading to DNA damage and apoptosis in immortal cholangiocyte cell lines [88]. However, an anti-apoptosis property of Triol and 3K4 was found in long term exposure to low doses of these chemicals in the same cell lines, suggesting that clonal expansion of such apoptosis-resistant cells may contribute to the genesis of cholangiocarcinoma [89]. Therefore, our studies demonstrated that the roles of etheno DNA adducts and oxystrols in cholangiocarcinoma can be considered as more than just biomarkers of oxidative stress induced lipid oxidation; they are also significant for carcinogenesis.

### 6.2. Carbonyl Protein in Cancers

Proteins are major biomolecules that drive cellular activities. Growing evidence suggests that oncoproteins, tumor suppressor proteins and oxidative stress-related proteins play significant roles in carcinogenesis. We previously demonstrated that oxidative stress induces carbonylated protein formations in liver fluke-associated cholangiocarcinoma tissues [27]. 2D-oxyblot revealed that the modified proteins were accumulated in the order of liver fluke-associated cholangiocarcinoma tissues > tumor-adjacent normal tissues > liver tissues from normal subjects. 2D-oxyblot and LC-MALDI-TOF/TOF analysis of separated proteins from the bile duct cancer tissues identified R50, K327, and P357 as carbonylated sites in serotransferrin, heat shock protein 70 protein 1 (HSP70.1), and alpha-1-antitrypsin (A1AT), respectively. Oxidative stress by inflammation caused the carbonylation of serotransferrin, which was coincident with iron accumulation, suggesting that the modified serotransferrin may lead to the accumulation and release of iron ion. The labile iron pool is a cellular source of iron ions available for the Fenton reaction, and may contribute to prolonged oxidative stress. HSP70.1 has been reported as protecting various cells from oxidative stress [90]. The carbonylation of HSP70.1 may induce its dysfunction of an antioxidant property, also resulting in prolonged oxidative stress. A1ATs, a glycoprotein, is a member of the serpins (serine protease inhibitors), inhibitors of a wide variety of proteases [91]. Serine proteases are produced by various cancer cells for digestion of the extracellular matrix together with matrix metalloproteinases (MMPs) for the induction of tumor invasion and angiogenesis [92]. Therefore, carbonylation of A1AT may lead to a conformational change and protein dysfunction, which could be involved in the progression of cholangiocarcinoma. Recently, we demonstrated that the serum levels of oxidized A1AT were increased in liver fluke infected subjects with advanced periductal fibrosis and in cholangiocarcinoma patients compared with normal subjects, suggesting that the oxidized A1AT can be used as a biomarker for predicting the risk of liver fluke-associated cholangiocarcinoma [93]. Moreover, high levels of plasma protein carbonyls were also found to increase the risk of breast cancers [94,95,96]. Our findings and previous studies suggest that dysfunction due to oxidized protein may lead to all stages of carcinogenesis: tumor initiation, promotion and progression.

### 6.3. DNA Damage in Inflammation-Related Cancers

Chronic inflammation induced by biological, chemical, and physical factors has been found to be associated with increased risk of cancer in various organs. We showed iNOS-dependent formation of 8-nitroguanine and 8-oxodG in cancer tissues and precancerous regions induced by infectious agents including *O. viverrini*, *Schistosoma haematobium*, *Helicobacter pylori* and human papilloma virus, and non-infectious agents such as asbestos fibers. These DNA lesions may induce carcinogenesis through the induction of mutation, genetic instability and epigenetic changes [97].

#### 6.3.1. DNA Damage in Parasite-Associated Cancers

**Figure 3:**
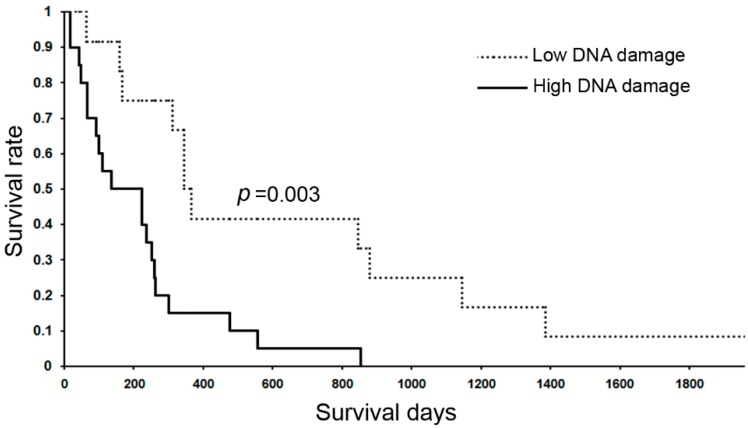
Kaplan-Meier analyses of 32 liver fluke-associated cholangiocarcinoma patients between high and low DNA damage formations in their tumor tissues. The levels of DNA damage were assessed by the double immunofluorescence method [107]. High formation of 8-oxodG and 8-nitroguanine was scored for high DNA damage accumulation in cholangiocarcinoma tissues (*n* = 20) and slight formation of DNA lesions was scored for low DNA damage accumulation in the tumor tissues (*n* = 10). Difference of survival was statistically analyzed by the log-rank test (*p* = 0.003, low *vs*. high DNA damage).

Liver fluke (*O. viverrini*) and blood fluke (*S. haematobium*) are classified as Group 1 carcinogens (IARC classification), being involved in cholangiocarcinoma and bladder cancer, respectively. DNA lesions were increased in *S. haematobium*-associated bladder cancers and *S. haematobium*-related cystitis in comparison with bladder cancer without the parasite infection and normal tissues [98]. 8-Nitroguanine, 8-oxodG and etheno adducts were formed and related with carcinogenesis both in animal and human models in *O. viverrini*-associated cholangiocarcinoma [99]. The detection of DNA lesions in urine is a potential biomarker for predicting the risk of cholangiocarcinoma in *O. viverrini-*infected subjects [85,100]. 8-Nitroguanine and 8-oxodG are mutagenic lesions that cause G→T transversion. Recently, exome sequencing identified G→T transversion mutation in several genes including ARID1A, KRAS and TP53 in liver fluke-related bile duct cancers, and functional analysis of ARID1A demonstrated a tumor suppressive function, suggesting the role of chromatin modulators in cholangiocarcinoma pathogenesis [60]. An immunohistochemical study for liver fluke-induced cholangiocarcinoma revealed that high formation of DNA damage lesions (8-nitroguanine and 8-oxodG) in cancer tissues is significantly correlated with poor prognosis (Log rank test; *p* = 0.003) as shown in Figure 3. Therefore, accumulating evidence and our reports suggest that inflammation-mediated DNA lesions play significant roles in not only tumor initiation, but also in tumor promotion and progression in parasite-related carcinogenesis.

#### 6.3.2. DNA Damage in Bacterial and Viral Infection-Related Cancers

*Helicobacter pylori* is the main cause of chronic gastritis, and a potential risk factor for gastric carcinoma [101]. Our previous study [45] demonstrated that levels of 8-nitroguanine and 8-oxodG in the gastric gland epithelium were significantly increased in gastritis patients with *H. pylori* infections compared to those without infections. A significant expression of proliferating cell nuclear antigen (PCNA) was also observed in gastric gland epithelial cells in patients infected with *H. pylori* in comparison to those not infected. Hepatitis B virus (HBV), hepatitis C virus (HCV) and Epstein-Barr virus (EBV) are group 1 carcinogens (IARC classification) related with hepatocellular carcinoma and nasopharyngeal carcinoma. Human papilloma virus (HPV) is also classified as a group 1 and a group 2A carcinogen, for high- and low-risk HPV types respectively, that induce cervical cancer. Inflammation induced by carcinogenic viral infections is proposed to play an integral role in the development of cancers. High-risk HPV types promote iNOS-dependent DNA damage, which leads to dysplastic changes and carcinogenesis [102]. Nuclear accumulation of epidermal growth factor receptor (EGFR) and activation of STAT3 by IL-6 play a key role in iNOS expression and resultant DNA damage, leading to EBV-related nasopharyngeal carcinoma [103,104]. This study also demonstrated that serum 8-oxodG could be a potential biomarker [103,104]. We observed a strong correlation between hepatic 8-oxodG staining and serum ferritin levels, suggesting that iron content to be a strong mediator of oxidative stress and iron reduction to reduce the incidence of hepatocellular carcinoma in patients with chronic HCV and HBV [105,106]. Therefore, it is plausible that ROS and RNS production during chronic carcinogenic bacterial and viral infections is the result of DNA damage in infected tissues, which leads to progressive tissue inflammation, and an increased risk of developing cancer.

#### 6.3.3. DNA Damage in Inflammation-Related Diseases and Cancers

Inflammation is a hallmark of carcinogenesis induced by asbestos, oral lichen planus (OLP), inflammatory bowel diseases (IBDs) and malignant fibrous histiocytoma (MFH). Asbestos is a carcinogenic agent (Group 1; IARC) that induces lung cancer. Among the different types of asbestos, crocidolite (blue asbestos) and amosite (brown asbestos) are more potent carcinogens than chrysotile (white asbestos) [108]. The staining intensities of 8-nitroguanine, iNOS and NF-κB were significantly higher in crocidolite-exposed mice than chrysotile-exposed mice [2]. Therefore, the formation of nitrative DNA damage could be one of the mechanisms responsible for the difference in carcinogenic potential between crocidolite and chrysotile. Our recent study demonstrated that 8-nitroguanine formation is associated with asbestos content in human lung tissues, suggesting that 8-nitroguanine serves as a biomarker that can be used to evaluate asbestos exposure and risk [109]. OLP is a chronic inflammatory mucosal disease [110] and a risk factor for oral squamous cell carcinoma (OSCC) [111]. The DNA lesions were observed in the order of OSCC > OLP > normal tissues, and were related with PCNA expression, suggesting that inflammation-mediated DNA damage and additional epithelial hyperplasia leading to cell proliferation promote oral carcinogenesis [112,113]. Epidemiological studies have shown that the incidence of colorectal cancer in IBD patients is greater than the expected incidence in the general population [114]. A high formation of DNA lesions was detected in clinical tissues of IBDs [97], and they correlated with an imbalance of T-cell function in an animal model [115]. These results suggest that nitrative DNA damage, as well as oxidative DNA damage, participates in colon carcinogenesis in patients with IBDs. Moreover, high 8-nitroguanine formation was significantly correlated with poor prognosis in MFH [116]. Therefore, 8-nitroguanine and 8-oxodG play important roles in inflammation-driven carcinogenesis and they can be used as a potential biomarker to predict the risk and prognosis of inflammation-related cancers.

### 6.4. DNA Damage in Stemness Cells of Inflammation-Related Cancers

**Figure 4:**
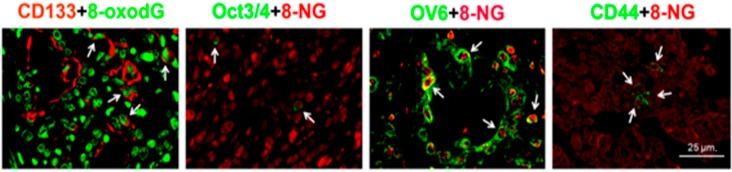
Double-immunofluorescence staining of stem/progenitor cell markers (CD133, Oct3/4, OV6 and CD44) and DNA lesions (8-oxodG and 8-nitroguanine (8-NG)) in cholangiocarcinoma tissues. White arrows indicate co-localization of DNA damage marker and stemness marker in cancer cells. Original magnification is ×400; Scale bar = 25 μm.

It has been suggested that stem/progenitor cells play roles in inflammation-related carcinogenesis [117,118]. Cells that have the ability to perpetuate themselves through self-renewal and to generate mature cells of a particular tissue by differentiation are defined as stem cells. Several mutations occur in cancer cells suggesting that cancer is a disease of genes, in which a cell ignores growth-limiting signals and forms a tumor that eventually leads to the death of the organism [119]. Not all tumor cells can participate in tumor evolution, and instead, this property is limited to a subset of cells, termed “cancer stem cells (CSCs)” which are also believed to be tumor initiating cells and resistant to oxidative stress, radiotherapy and chemotherapy [119,120]. Many proteins have been proposed as stemness markers in various types of cancers [121]. CD133, Oct3/4, CD44 and oval marker 6 (OV6) were proposed to be stemness markers in liver fluke-related cholangiocarcinoma [107]. Oct3/4 and CD44v6 were also used as stemness markers in bladder cancers [98,122]. Oct3/4 and CD44 expressions were reported to play important roles in the induction of antioxidant defense systems through thioredoxin and glutathione [123,124,125]. Moreover, several lines of evidence also suggested that CSCs have lower intracellular ROS contents and more resistance to oxidative stress and radiotherapy than non-CSCs, which may be due to the increased expression of antioxidant systems [126]. However, our recent publications demonstrated that DNA lesions (8-oxodG and 8-nitroguanine) were formed in CSCs that had Oct3/4 expression in *S. haematobium*-associated bladder cancer, and CD133, Oct3/4, oval marker 6 (OV6) and CD44 expressions in *O. viverrini*-associated cholangiocarcinoma [121]. Additionally, DNA lesions were significantly increased in *O. viverrini*-associated cholangiocarcinoma patients with high expressions of CD133 and/or Oct3/4 in their tumor tissues, whereas there were no significant differences of DNA lesions, in patients, between CD44 and/or OV6-positive and negative tumor tissues [101]. Interestingly, Oct3/4 was highly expressed in *S. heamatobium*-associated bladder cancers and was significantly related with high DNA damage formation whereas CD44v6 was related with low DNA damage formation in bladder cancers without the parasite infection [98,122]. Figure 4 shows the co-localization of stemness markers including CD133, Oct3/4, oval marker 6 (OV6) and CD44 with DNA lesions in clinical cholangiocarcinoma tissues. These studies strongly indicated that in some cases the redox status of the stem cells fails to protect the cells from oxidative damage which contributes to DNA damage, mutation and genetic instability of the CSCs resulting in tumor progression with a poor prognostic outcome.

### 6.5. Oxidative Stress and DNA Methylation

Epigenetics including DNA methylation and histone modification is a kind of gene regulation system without genetic sequence change. In general, DNA hypermethylation at the gene promoter region and histone deacetylation cause gene silencing. It has been suggested that oxidative stress is involved in DNA hypomethylation. Since oxidative stress depletes glutathione, and thereafter the increase of its production decreases the cellular levels of *S*-adenosylmethionine (SAM), it contributes to the lack of DNMTs substrate, leading to genome-wide DNA hypomethylation [127]. DNMTs catalyze the transfer of the methyl donor group from SAM to cytosine nucleotide at the CpG site. ROS-induced DNA damage such as 8-oxodG and O^6^-methylguanine and point mutations can interfere with the binding of DNMTs and therefore can lead to hypomethylation by means of inhibiting methylation of adjacent cytosine molecules [128]. In contrast, ROS are found to induce DNMT1 expression and activity with MBD4 cooperation and through a mitochondrial ROS-JNK-DNMT1 pathway [129,130]. In addition, IL-6, an important proinflammatory cytokine, has been reported to increase DNA hypermethylation through IL-6-mediated Janus kinase (JAK)/STAT3 pathways and AKT kinase via activation of DNMT1 expression and to increase its activity [131,132,133]. We demonstrated IL-6 modulated iNOS expression via STAT3 and EGFR in EBV-associated nasopharyngeal carcinoma [105]. Epigenetic silencing of tumor suppressor genes plays an important role in EBV-associated neoplasia [134]. We, and our colleagues, have found promoter hypermethylation in several candidate tumor suppressor genes for EBV-associated nasopharyngeal carcinoma [135,136,137,138,139]. Therefore, accumulating evidence suggests that oxidative stress is involved in aberrant methylation, both genome-wide DNA hypomethylation and promoter hypermethylation, although detailed mechanisms remain to be clarified.

## 7. Conclusions: Oxidative Stress and Its Significant Roles in Neurodegenerative Diseases and Cancer

**Figure 5:**
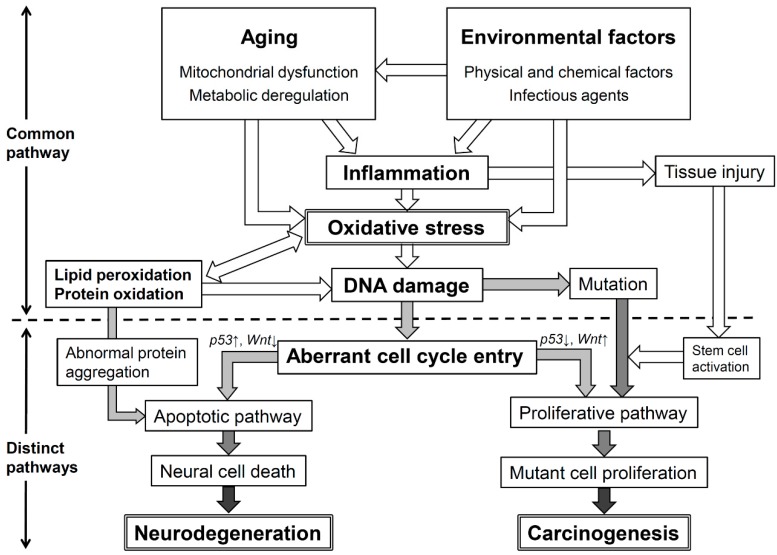
Roles of oxidative stress in neurodegenerative diseases and cancer.

Aging is a multifactorial process derived from an interaction between genetic and environmental factors. Aging is accompanied by an up-regulation of inflammatory responses, and inflammatory changes are common to many age-related diseases such as neurodegenerative diseases and cancer [140]. Environmental factors can cause metabolic changes in humans that either increase the production of ROS/RNS or decrease the antioxidant production with increased lipid peroxidation, protein and DNA oxidation [141]. Oxidative stress induces the formation of lipid peroxidation leading to prolongation of oxidative stress via the propagating chain reaction [142]. Oxidized proteins accumulate in cells via aggregations, protein aggregates cause more mitochondrial damage, and damaged mitochondria can further induce protein damage [143]. Moreover, reactive species damage DNA, which may lead to aberrant cell cycle entry, and further to differential regulation of common genes such as *p53* and *Wnt* in neurodegenerative diseases and cancer [144]. As shown in Figure 5 (distinct pathway), the striking differences between post-mitotic neurons and regular mitotic cells provide insight into the inverse association between cancer and neurodegeneration, that is, apoptosis of neural cells (up-regulation of *p53* and down-regulation of *Wnt*) and proliferation of cancer cells (down-regulation of *p53* and up-regulation of *Wnt*) [144]. Additionally, tissue injury can activate normally quiescent adult liver stem cells that thereby become a potential target cell population in many cancers. When several carcinogenic events such as mutation and epigenetic changes occur in stem cells under oxidative stress, the cells may acquire the properties of cancer stem cells. Nevertheless, age- and environment-related inflammation and oxidative stress may be a common initiating event for both neurodegeneration and carcinogenesis, as shown in Figure 5 (common pathway). Overall, the common pathway could be a target for developing chemopreventive and therapeutic strategies against oxidative stress in neurodegenerative diseases and cancer.
